# The effects of music and virtual reality on pain and anxiety during central venous port implantation: a randomised clinical trial

**DOI:** 10.1038/s41598-026-42184-w

**Published:** 2026-03-07

**Authors:** Abdelmalek Ghimouz, Sylvain Dureau, Matthieu Carton, Alexandra Gomola, Ziad Fadel, Kim Khanh Thuong, Jane Muret

**Affiliations:** 1 Department of Anaesthesia, Intensive Care, and Pain, Institut Curie, 26, rue d’Ulm, Paris, 75005 France; 2 Departement of Statistics. Institut Curie Paris, 26, rue d’Ulm, Paris, 75005 France; 3 Departement of Anaesthesia, Intensive care, and Pain. Institut Curie, 35, Rue Dailly, Saint Cloud, 92210 France; 4 Departement of Anaesthesia. Institut Gustave Roussy, 114, Rue Edouard Vaillant, Villejuif, 94800 France

**Keywords:** Central venous port, Pain, Anxiety, Music, Virtual reality, Diseases, Health care, Medical research, Signs and symptoms

## Abstract

The value of music (MUS) and virtual reality (VR) in reducing pain or anxiety during central venous port implantation (CVPI) is controversial. We conducted a randomised multicenter controlled trial in 127 patients who received either MUS (38) or VR (38) during CVPI compared to standard (STAND) group (51). The primary outcome was a co-criterion related to pain or anxiety experienced during CVPI assessed using a Numerical Rating Scale. Pain and anxiety were considered independently. The secondary outcomes were the tolerance of the MUS and VR devices, patient satisfaction, and the correlation between mean pain scores and Analgesia Nociceptive Index scores (ANI). There were no differences in pain or anxiety between MUS and STAND. Mean pain was 3.3 ± 2.2 (SD) vs. 3.3 ± 2.6; (*P* > 0.9) and mean anxiety was 4.4 ± 2.8 vs. 4.2 ± 3.1; (*P* = 0.6). There were no differences in pain or anxiety between VR and STAND. Pain was 3.6 ± 2.3 vs. 3.3 ± 2.6; (*P* = 0.5) and anxiety was 3.2 ± 2.3 vs. 4.2 ± 3.1; (*P* = 0.11). The MUS and VR devices were well tolerated. Patients were very satisfied. No correlation was observed between pain scores and ANI scores in the three groups. The use of MUS or VR during CVPI had no beneficial effect on reducing pain or anxiety.

Trial resgistration ClinicalTrials.gov: NCT04804735; Registred on 16/03/2021.

Central venous port implantation (CVPI) has beneficial effects and excellent acceptance for patients suffering from cancer^1^. In most cases, CVPI is performed under local anesthesia (LA). During CVPI, patients may experience discomfort due to pain and anxiety, which may lead to the discontinuation of the procedure with a request for general anesthesia. This cancellation may further escalate the patient’s anxiety. Pain may be related to the needle prick, the LA injection, and central vein cannulation with the dilator. Some authors have proposed the use of intravenous sedatives and analgesics^2–4^. The use of sedatives or analgesics during surgical procedures requires additional nursing staff, which represents a financial cost that must be taken into consideration. The use of ultrasound guidance for internal jugular vein (IJV) cannulation leads to a time reduction of the CVPI^5^. IJV cannulation results in less pain than subclavian access^6^. The use of ultrasound guidance and the internal jugular route instead of the subclavian route represents a major advance. Non-pharmacological tools such as music (MUS) and virtual reality (VR) have recently been marketed to manage chronic pain, or pain and anxiety during medical care procedures with good results in terms of reducing pain and anxiety^7–10^. Studies evaluating the use of MUS or VR during CVPI have yielded controversial results, both in terms of pain reduction and anxiety reduction. The studies in question were single-center, which limits their impact^11,12^. We therefore conducted a multicenter study simultaneously comparing a MUS group and a VR group to a standard (STAND) group during CVPI. We hypothesized that the use of MUS or VR during CVPI via the internal jugular vein under local anesthesia could reduce pain or anxiety compared to a STAND group. We did not use sedatives or analgesics in order to avoid any interference with the effects of music or virtual reality.

## Materials and methods

### Ethics approval and study design

This prospective, controlled, randomised and multicenter trial was conducted in accordance with the Declaration of Helsinki and conformed to the Consolidated Standards of Reporting Trials guidelines (CONSORT). The study protocol was registered at ClinicalTrials.gov (NCT04804735) on 16/03/2021 and approved by the Institutional Review Board (Institut Curie, Paris, France) and by the ethics committee of Ile de France on 27/05/2021 (CPP Ile de France VIII; President Dr F. Le Mercier; reference number, 210109). The study was conducted in accordance with the original protocol without any change, from May 2022 to August 2023 in three Cancer Institutes in France (Institut Curie, Paris; Institut Curie, Saint Cloud; Institut Gustave Roussy, Villejuif) and was completely financed by public funds (Région Île-De-France). Patients were informed at least 24 h before CVPI. Informed consent was obtained from all the study participants. The patients were allowed to withdraw their consent at any time.

The inclusion criteria were as follows: patients aged 18–85 years, treated for cancer and requiring the placement of a CVPI under LA for chemotherapy, who had not received any premedication for anxiety, were affiliated with social security, and had no allergy to lidocaine.

The exclusion criteria were as follows: patients who had already participated in this study, patients who did not speak French, patients deprived of their liberty or under legal guardianship or conservatorship, psychiatric illness preventing communication, deafness or visual impairment, patients taking beta-blockers that interfere with the monitoring of the Nociceptive Analgesia Index, moderate or severe uncontrolled chronic pain that could interfere with the acute pain caused by catheter placement, tracheotomy, low cervical tumor, bilateral low cervical lymphadenopathy, unilateral or bilateral compression or stenosis of the IJV, thrombosis of the IJV or subclavian vein, patients on anticoagulants or with hemostasis disorders, claustrophobia that may lead to rejection of the VR mask or music headphones, pregnant and breastfeeding women.

### Randomisation

Before starting the procedure, the investigator checked the inclusion and exclusion criteria and filled in the electronic Case Report Form (eCRF) for sex, weight, height, tumor location, TNM classification, and indication for chemotherapy (neoadjuvant, adjuvant, or metastasis). Randomisation was performed using an online computer program. Patients were randomised into 3 groups (MUS, VR, and STAND) according to computer-generated random numbers. In each group, randomisation was stratified according to anticipated difficulties with CVPI and reported anxiety prior to surgery. The CVPI procedure was considered potentially difficult in cases of left internal jugular vein puncture, obesity, or a short and stocky neck. Anxiety > 3 prior to surgery was used to classify patients into the anxious group, and a value ≤ 3 was used to classify patients into the non-anxious group^13^.

## Intraoperative management

After randomisation, patients were admitted to the operating room. No sedatives or analgesics were administered before or during surgery. Before the start of the procedure, headphones were placed over the ears in the MUS group, and masks were placed over the eyes and headphones over the ears in the VR group. The MUS and VR devices were used throughout the surgical procedure and removed at the end of the procedure. In the STAND group, the anesthetist was able to converse with patients as they wished. CVPI was performed under LA with a mixture of lidocaine 1% (20 ml) and sodium bicarbonate (4 ml) by an experienced anesthetist via the right or left IJV under ultrasound guidance using the Seldinger technique. The correct position of the catheter at the entrance of the right atrium was confirmed using intraoperative fluoroscopy. All patients were connected to Analgesia Nociceptive Index monitor (ANI). Four operative times have been defined for the ANI: T0 (time of skin disinfection), T1 (time of subcutaneous injection of LA), T2 (time of cannulation of IJV with the dilator), and T3 (time of skin suture). During the procedure, the nurse pressed the event button on the ANI monitor to record T0, T1, T2, and T3 in a file. The duration of the surgical procedure ranges from skin disinfection to skin suturing. This duration was recorded for each patient in the three groups. At the end of the procedure, the MUS, VR, and ANI devices were disconnected. Within 15 min of surgery, average scores for pain and anxiety experienced during surgery, tolerance of MUS and VR devices, and patient satisfaction were collected in the recovery room by a nurse or anesthetist who had not participated in the operation in order not to influence patient responses. All patients were allowed to return home after the procedure.

## Music device

The music device is marketed by the company (MUSIC CARE^™^, France). Patients selected orchestral music from a tablet according to their taste. After headphone installation, the volume of music was adjusted for the patient’s convenience. We used “U” musical relaxation. At the beginning of the sequence, the rhythm of the music was high. The rhythm gradually decreased, inducing relaxation during CVPI. At the end of the procedure, the rhythm gradually increased and remained below the starting frequency. The musical sequence lasted 30–45 min and could be shortened or extended by the nurse depending on the duration of the surgical procedure. The patient could ask to stop the music or ask a question at any time.

## Virtual reality device

The VR mask was marketed by the company (OnComfort-Sedakit^™^, Belgium). The film “AQUA” was used for hypnotic relaxation through an underwater journey. The film suggested self-hypnosis without the intervention of a healthcare professional. During the first part, patients were asked to become aware of different parts of their body and their breathing. In the second part, the shuttle dove for underwater adventure. Patients encountered a whale that they had to follow during immersion. They were suggested to synchronize their breathing with undulating movements of the whale’s tail during the hypnotic relaxation phase. At the end of the journey, the shuttle rose to the surface and patients regained full consciousness. This scenario lasted between 30 and 45 min and could be shortened or extended by the nurse depending on the duration of the surgical procedure. Patients could ask to stop the film or ask a question at any time.

## Analgesia nociceptive index

The ANI is a monitor marketed by MDoloris (ANI, MDoloris Medical Systems, Loos, France). The monitor continuously displayed ANI values ​​ ranging from 0 to 100. This monitor measured the balance of nociception and antinociception on a scale from 0 (maximum nociception = predominance of the sympathetic nervous system) to 100 (complete analgesia = predominance of the parasympathetic nervous system), making a distinction between appropriate and inappropriate antinociception in anesthetised adult patients^14^.

### Primary and secondary objectives

The primary objective of this study was to assess the impact of MUS and VR on pain or anxiety compared to STAND. Pain and anxiety were considered independently. We considered that an improvement in the management of pain or anxiety could justify the use of MUS or REV devices. Mean pain and anxiety were rated on a Numerical Rating Scale (NRS) from 0 (no pain or anxiety) to 10 (worst pain or anxiety). The secondary objectives were as follows: to assess tolerance to MUS and VR devices (feelings of dizziness, suffocation, or nausea) using closed-ended questions with “yes” or “no” answers; to assess patient satisfaction on a NRS ranging from 0 = no satisfaction to 10 = best satisfaction experienced; to assess the correlation between mean pain scores and ANI scores. Primary and secondary outcomes were performed on the intent-to-treat population, defined as all randomised patients.

### Statistical analysis

The primary outcome was a co-criterion of “pain or anxiety”. A 2-point reduction in pain scores or in anxiety scores was considered acceptable for detecting clinically meaningful improvement^15,16^. Based on the results of previously reported pain and anxiety scores, the expected clinical difference between the MUS and STAND groups or between VR and STAND groups was 2 points using NRS with a standard deviation of 2.5^4,16^. With a type 1 error equal to 2.5% (Bonferroni correction for test multiplicity due to the co-criterion) and a power of 90% for each of the two criteria, 37 patients were required in each of the three groups of the trial. However, the STAND was the reference group compared with each of the two experimental groups (MUS and VR). To account for this double comparison with the same STAND and to maintain an overall of risk of 5% for the analysis of the co-primary endpoint, the number of subjects in the single reference group (STAND) was increased by a Root-Square factor (g-1), where g corresponds to the total number of groups (g = 3 in this trial)^17^. Thus, 51 patients were included in the STAND and 38 in each of the MUS and VR.

Mean pain and anxiety scores during CVPI were compared between MUS and STAND groups and between VR and STAND groups using linear models adjusted for randomisation stratification factors (expected difficulty and level of anxiety prior to CVPI). Satisfaction was compared between each of the three groups using the Kruskal-Wallis rank sum test or Fisher’s exact test. All the tests were performed at a threshold of 5%. Analyses were performed using R software (4.4.2 version). The mean ANI score was correlated with the mean pain score, and the correlation coefficients were estimated with 95% confidence intervals.

## Results

Among the 128 patients who agreed to participate in this trial, one had no data concerning inclusion and non-inclusion criteria and was therefore not randomised.

**Fig. 1:**
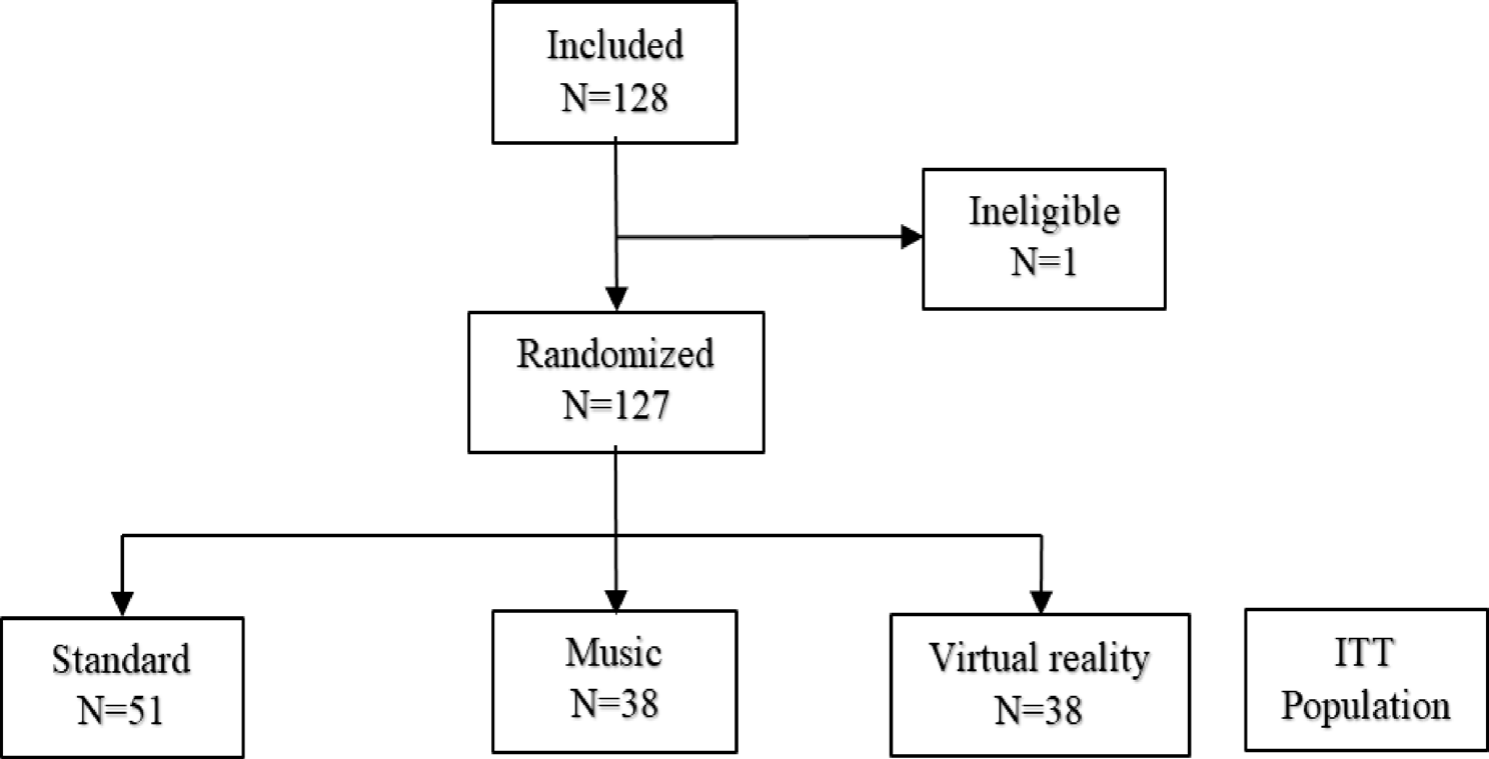
Flow chart.

A total of 127 patients were included in the trial (Fig. 1, flow chart). All patients met the inclusion and exclusion criteria. They were randomised into three groups: 38 patients in the VR group, 38 in the MUS group, and 51 in the STAND group. Five patients (four in the MUS group and one in the VR group) were unable to benefit from the randomly assigned intervention because the batteries in the MUS and VR devices were low; they received the intervention in the STAND group. These patients were analysed according to their randomisation group in accordance with the intention-to-treat principle. Data on pain and anxiety during the procedure were missing for two patients: one in the STAND group and one in the MUS group.

**Fig. 2:**
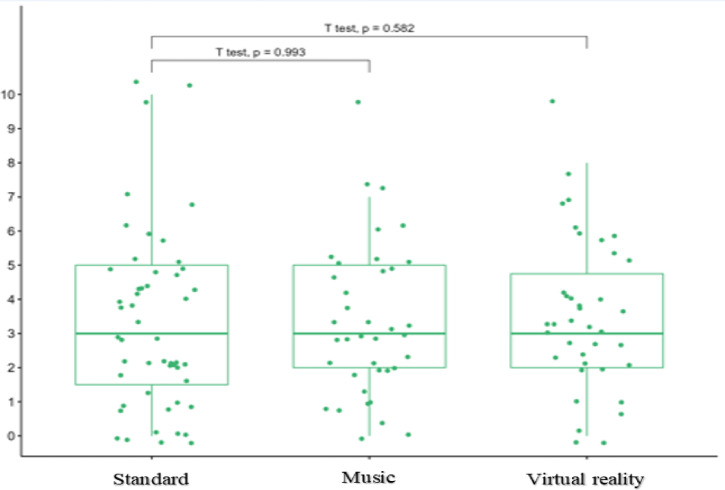
Pain experienced during central venous port implantation.

**Fig. 3:**
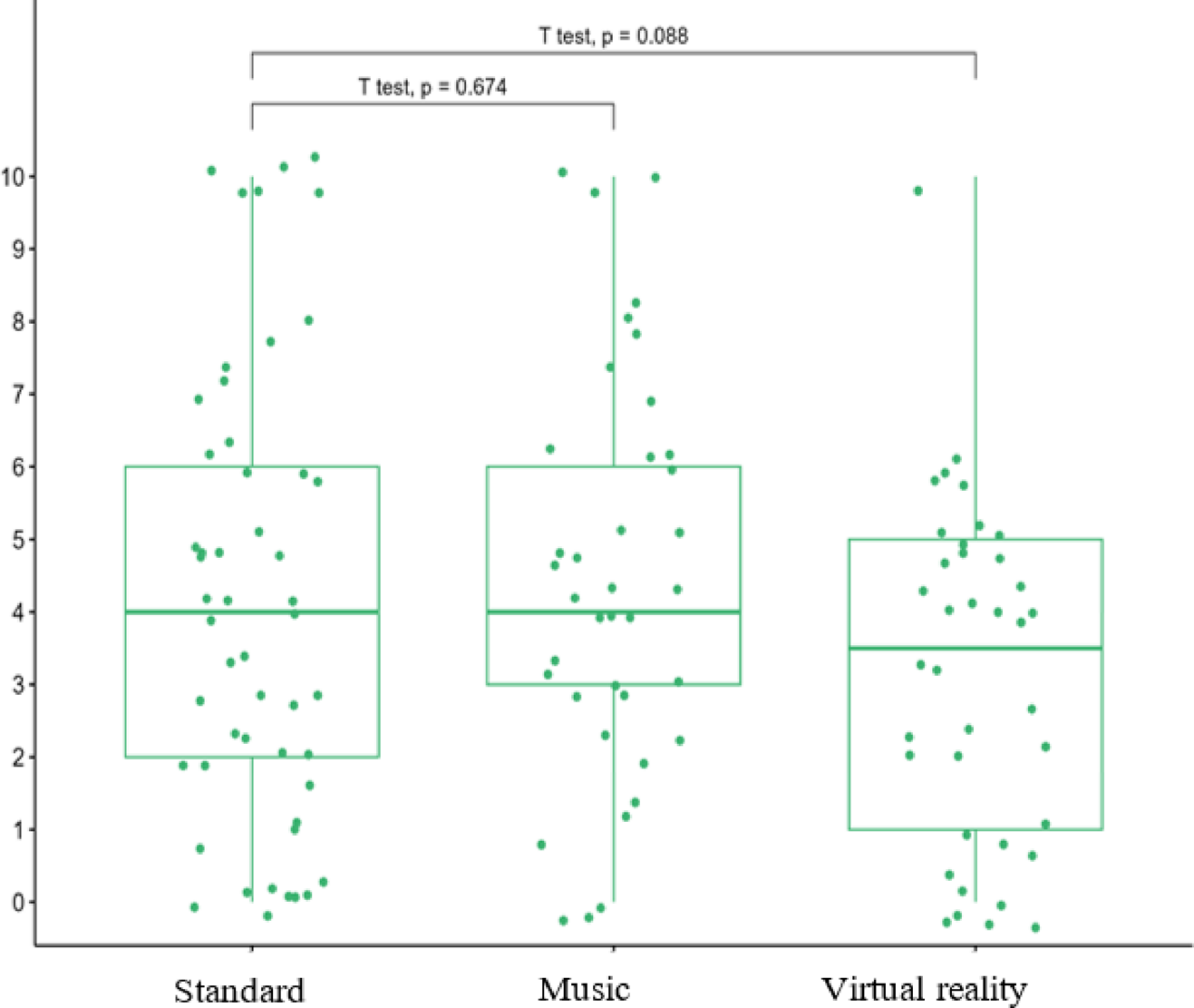
Axienty experienced during central venous port implantation.

**Fig. 4:**
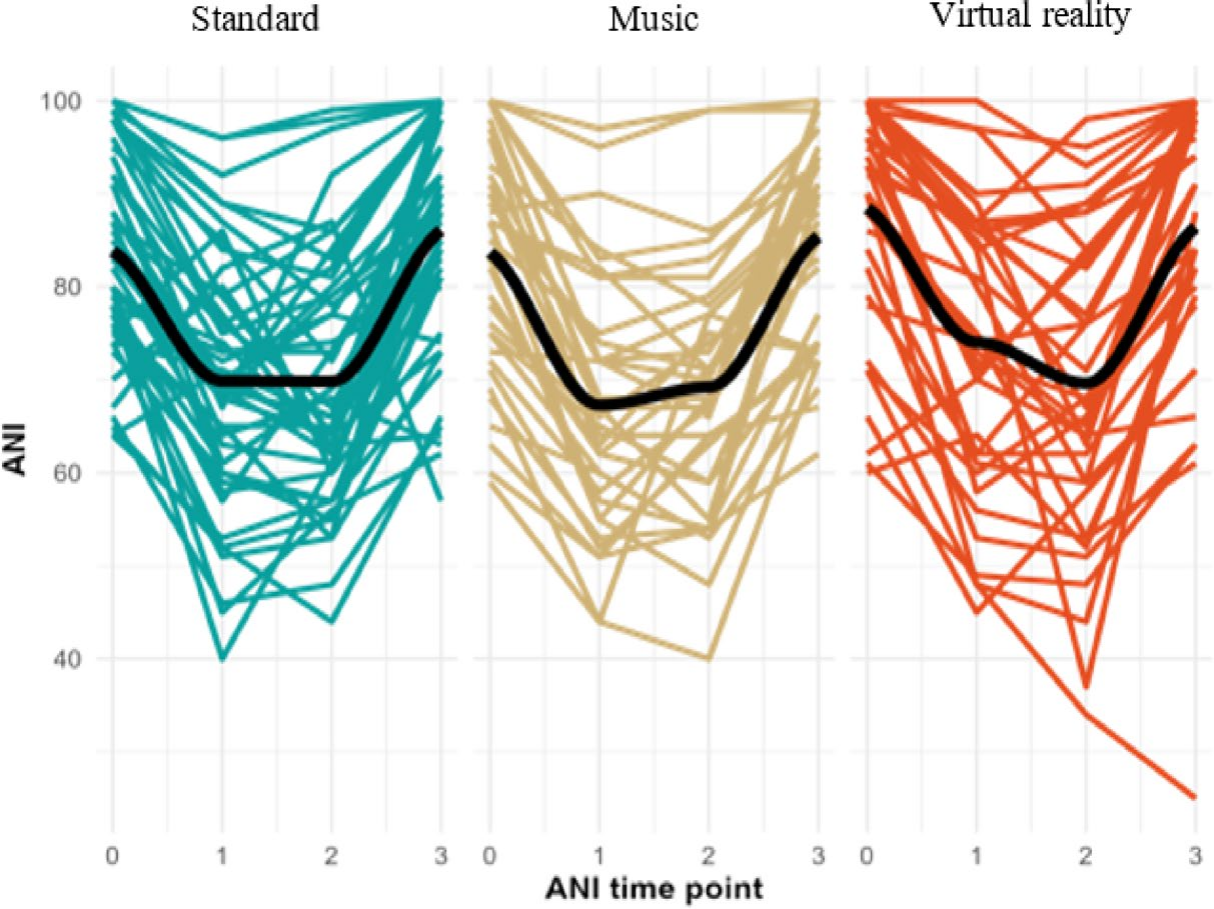
Evolution of analgesia nociceptive index (ANI) at 4 measurements points.

The randomised patients had similar characteristics in all three groups (Table 1). The average duration of surgical procedures in the MUS, VR and STAND groups was, respectively 32 ± 10.2 min; 34.5 ± 8.2 min; 30 ± 9.2 min. There was no difference between the three groups. The pain and anxiety scores experienced during the procedure are schown in Table 2 (Figs. 2 and 3). There were no differences in pain or anxiety between MUS and STAND group. There were no differences in pain or anxiety between VR and STAND group. Based on the predictable difficulties of CVPI or anxiety before the surgical procedure, we did not find a significant difference between patients in the three groups. The tolerances of the MUS and VR devices were similar in both groups: 1 case of dizziness and 2 feeling of suffocation in MUS group; 3 dizziness, 1 nausea and 3 feeling of suffocation in VR group (Table 3). Patients were very satisfied with the CVPI, with no difference between the three groups (Table 4). There were no differences in ANI scores at different time points between the 3 groups (Table 5; Fig. 4). We found no correlation between the pain scores and the ANI scores at T1 and T2 in any of the 3 groups.

**Table 1:** Patient characteristics.

Characteristics	*N* 127	STAND *N* = 51	MUS *N* = 38	VR *N* = 38
Age (yr), mean (SD)	56 ± 13	57 ± 15	54 ± 12	57 ± 12
Females, n/total N (%)	111/127 (87%)	44/51 (86%)	35/38 (92%)	32/38 (84%)
Age (yr) at diagnosis, Mean (SD)	55.6 ± 13	57 ± 15	54 ± 12	56 ± 12
Breast cancer n/total (%)	83/127 (65%)	28/51 (60%)	29/38 (76%)	26/38 (68%)
Anxious	90/127 (70%)	37/51 (73%)	27/38 (71%)	26/38 (68%)
Not anxious	37/127 (29%)	14/51 (27%)	11/38 (29%)	12/38 (32%)
Difficulties expected	61/127 (48%)	25/51 (49%)	19/38 (50%)	17/38 (45%)
No difficulties expected	66/127 (52%)	26/51 (51%)	19/38 (50%)	21/38 (55%)

Data are displayed as mean ± SD, n/total *N* (%). SD = standard deviation.

STAND , Standard group; MUS, Music group; VR ,  Virtual reality group.

**Table 2:** Primary outcomes.

		Pain		Anxiety	
	*N*	Mean (SD)	*p*-value^*^	Mean (SD)	*P* value^*^
Stand	51	3.3 ± 2.6		4.2 ± 3.1	
MUS	38	3.3 ± 2.2	> 0.9	4.4 ± 2.8	0.6
VR	38	3.6 ± 2.3	> 0.5	3.2 ± 2.3	0.11

^*^Wald test, adjusted for stratification factors. Numerical rating scale was used to asses pain and anxiety during central port implantation (0 = no pain or anxiety and 10 = worst pain or anxiety). Data are displayed as mean ± SD. We found no significant differences beteween the three groups. SD = standard deviation.

**Table 3:** Tolerance of devices.

		Group		
Characteristic	*N*	MUS *N* = 38	VR *N* = 38	*p*-value^*^
Feeling of dizziness	73			0.6
No		35.0 (97.2%)	34.0 (91.9%)	
Yes		1.0 (2.8%)	3.0 (8.1%)	
Unknown		2	1	
Feeling of nausea	73			> 0.9
No		36.0 (100.0%)	36.0 (97.3%)	
Yes		0.0 (0.0%)	1.0 (2.7%)	
Unknown		2	1	
Sensation of suffocation	73			> 0.9
No		34.0 (94.4%)	34.0 (91.9%)	
Yes		2.0 (5.6%)	3.0 (8.1%)	
Unknown		2	1	

*Wilcoxon rank sum test; Pearson’s Chi-squared test; Fisher’s exact test. We found no significant differences beteween the music group and virtual reality group.

**Table 4:** Patient satisfaction.

		Group			
Characteristic	*N*	Standard *N* = 51	Music *N* = 38	Virtual reality *N* = 38	*p*-value^*^
Patient satisfaction	124				0.3
Mean ± SD		9.3 ± 1.2	8.8 ± 1.8	8.9 ± 1.6	
Unknown		1	1	1	

Numerical rating scale was used to asses patient satisfaction with central venous port implantation (0 = dissatisfied patient and 10 = very satisfied patient). Patient satisfaction is expressed as mean ± standard deviation. We found no significant differences beteween the three groups.

^*^Kruskal-Wallis rank sum test; Fisher’s exact test.

**Table 5:** Scores of ANI at T0, T1, T2 and T3.

		Group			
Characteristic	*N*	Standard *N* = 51	Music *N* = 38	Virtual reality *N* = 38	*p*-value^*^
ANI at T0	122				0.066
Mean ± SD		83.8 ± 10.7	83.7 ± 12.8	88.4 ± 12.7	
Unknown		1	3	1	
ANI at T1	122				0.094
Mean ± SD		69.8 ± 13.1	67.3 ± 13.5	74.0 ± 15.2	
Unknown		1	3	1	
ANI at T2	121				> 0.9
Mean ± SD		69.8 ± 13.1	69.2 ± 13.3	69.6 ± 16.7	
Unknown		1	4	1	
ANI at T3	120				0.6
Mean ± SD		86.0 ± 12.1	85.4 ± 10.9	86.4 ± 16.1	
Unknown		2	4	1	

*Kruskal-Wallis rank sum test. ANI = Analgesia nociceptive index. Scores of ANI are expressed as the mean ± standard deviation. T0 = time for skin disinfection; T1 = time for subcutaneous injection of local anesthetic; T2 = time for introduction of tunneler into internal jugular vein; T3 = time for skin suture. We found no significant differences beteween the three groups.

## Discussion

This study, designed to directly and simultaneously evaluate the benefit of MUS or VR compared to a STAND group during CVPI, found no difference between the three groups, even in patients with predictable difficult insertion criteria or in anxious patients. The results would have been similar if the 4 patients in the MUS group had received music as planned and the patient in the VR group had received virtual reality device as planned. Unlike other single-center studies, the fact that our study was multicenter strengthens our results. Studies have shown that using ultrasound for IJV cannulation reduces the duration of the surgical procedure⁵. Other studies have shown that cannulation of the IJV is less painful than the subclavian approach ⁶. We have adopted the ultrasound-guided approach for the IJV in our daily practice, which greatly improves patient and clinician comfort. We were therefore already familiar with this practice when we conducted this study. Our practice of cannulation the IJV under ultrasound control is in accordance with the recommendations published in 2002 by the National Institute for Health and Care Excellence (NICE)^18^. One study found an average pain level of 5 when using plain lidocaine for skin infiltration^19^. Pain resulting from skin infiltration with lidocaine solution can be reduced to an average of 1.5 by adding sodium bicarbonate^20^. That is why we added sodium bicarbonate to the lidocaine solution in our daily practice and to conduct this study. Despite this, the average pain scores were greater than 3 in all three groups. The pain was likely due to the venous cannulation rather than the subcutaneous injection of the LA. To design this study, we chose a 2-point reduction in pain or anxiety in the MUS and VR groups compared to the STAND group, as this difference is generally considered clinically significant^15,16^. Contrary to our expectations, we observed the same levels of pain and anxiety in all three groups. We probably would not have noticed a difference either if we had chosen a difference of 1-point between the MUS and VR groups compared to the STAND group. MUS and VR are used in various settings to manage pain and anxiety during medical procedures with good results compared to control groups. However, the reduction of pain or anxiety through the use of MUS or VR during central catheter placement remains controversial. One single-center study found a significant reduction in pain in the MUS group compared to the control group (3.14 versus 3.86) during CVPI via the subclavian route under LA^12^. This reduction of less than 1 point remains clinically insignificant^15^. Furthermore, in this study, the music was played through loudspeakers. The caregivers assessing the patients listened to the same music, which likely influenced the patients’ responses^12^. Another single-center study found a significant decrease in anxiety and a significant decrease in heart rate in the MUS group compared to the control group during central catheter cannulation^21^. In contrast to this study, Schaal et al. found no difference between the MUS group and the control group in terms of anxiety, but observed a significant decrease in blood pressure and heart rate compared to baseline values ​​in the MUS group^16^. Regarding the use of VR, several clinical trials have shown that its use in patients suffering from burns, trauma, receiving dental care, or undergoing peripheral venous cannulation resulted in a significant reduction in pain and anxiety^10,22^. One recent meta-analysis confirmed the value of VR in managing acute pain during medical care^23^. Unfortunately, few studies have assessed pain and anxiety during CVPI using VR and the reduction of pain or anxiety through the use of VR during CVPI remains uncertain. A single-center clinical trial showed a significant reduction in anxiety and pain in the VR group compared to the control group after CVPI managed under LA^24^. In this study, intraoperative anxiety and pain were not assessed. Anxiety assessed after the CVPI was compared to pre-operative anxiety, and pain at the incision site was assessed 4 h after the CVPI. Therefore, comparison with other studies is difficult. Consistent with our findings, Steinkrauss et al., in a single-center randomized controlled trial evaluating perioperative pain and anxiety during subclavian port implantation, found no difference between the VR group and the control group^11^. The NRS has been validated for pain assessment in oncology^25^. The NRS and STAI scales allow anxiety to be measured with a good correlation between the two scales^13,26^. That is why we used the NRS in our study to quickly assess both pain and anxiety within 15 min of the CVPI.

The clinical trials do not always find correlations between hemodynamic or cortisol variations and anxiety or pain^12,16^. In our study, we tested the ANI monitor as a secondary outcome to objectively assess pain intensity throughout the surgical procedure. The ANI allows the objective measurement of nociceptive stimuli during surgery under general anesthesia. Following a pilot study that demonstrated a good correlation between ANI results and pain assessed by the NRS in pregnant women during labour^27^, we used ANI to objectively assess pain during CVPI. We did not find any correlation between ANI scores and pain scores. The sympathetic nervous system stimulated by the stress during CVPI may have interfered with the ANI results. We concluded that ANI is not a suitable device for monitoring pain in case of CVIP under LA^28^.

### Limitations

The main limitation of the study is that it was an open-label trial. It might have been necessary to compare, in a double-blind study, a group receiving hypnotic music with a group receiving ordinary music, and a group receiving a mask with a hypnotic film with a group receiving an ordinary film. Another limitation is that, unlike the MUS group where patients could choose the music they wanted, in the VR group we used the only film we had available. In this study, some patients have been frustrated at not having music or VR. This may have interfered with the results. We thought that the ANI could help us objectively assess pain in these non-anesthetised patients as showed in one pilot study. Unfortunately, the ANI which is not used in our practice during the CVPI, made the study more complex, without providing any benefits.

In conclusion, although our results did not demonstrate a reduction in pain or anxiety in patients undergoing CVPI using MUS or VR, the study highlights the feasibility, safety, and patient acceptability of non-phamacological interventions in oncologic setting. This negative trial should not rule out the use of music or virtual reality devices for pain and anxiety management during painful medical procedures other than CVPI.

## Data Availability

The data supporting the conclusions of this study are not publicly available due to ethical and confidentiality restrictions, but may be obtained from the corresponding author upon reasonable request.

## References

[1] Niederhuber, J. E. et al. Totally implanted venous and arterial access system to replace external catheters in cancer treatment. *Surgery* **92** (4), 706–712 (1982). 7123491

[2] Burlacu, C. L., McKeating, K. & McShane, A. J. Remifentanil for the insertion and removal of long-term central venous access during monitored anesthesia care. *J. Clin. Anesth.* **23** (4), 286–291 (2011). 21663812 10.1016/j.jclinane.2010.12.007

[3] Bosch, F. H. & Schiltmans, S. K. Stepwise sedation is safe and effective for the insertion of central venous catheters. *Neth. J. Med.* **62** (1), 18–21 (2004). 15061228

[4] Samantaray, A. & Rao, M. Additional analgesia for central venous catheter insertion: A placebo controlled randomized trial of dexmedetomidine and fentanyl. *Crit. Care Res. Pract.*10.1155/2016/9062658 (2016). 27200187 10.1155/2016/9062658 PMC4856885

[5] Hind, D. et al. Ultrasonic locating devices for central venous cannulation: meta-analysis. *BMJ* **327**10.1136/bmj.327.7411.361 (2003). 10.1136/bmj.327.7411.361 PMC175809 12919984

[6] Plumhans, C. et al. Jugular versus subclavian totally implantable access ports: Catheter position, complications and intrainterventional pain perception. *Eur. J. Radiol.* **79** (3), 338–342 (2011). 20227211 10.1016/j.ejrad.2009.12.010

[7] Guétin, S. et al. The effects of music intervention in the management of chronic pain: a single-blind, randomized, controlled trial. *Clin. J. Pain*. **28** (4), 329–337 (2012). 22001666 10.1097/AJP.0b013e31822be973

[8] Spiegel, B. et al. Virtual reality for management of pain in hospitalized patients: A randomized comparative effectiveness trial. *PLoS One* **14** (8), (2019). 10.1371/journal.pone.0219115 PMC6693733 31412029

[9] Hole, J., Hirsch, M., Ball, E. & Meads, C. Music as an aid for postoperative recovery in adults: A systematic review and meta-analysis. *Lancet* **386** (1040), 1659–1671 (2015). 26277246 10.1016/S0140-6736(15)60169-6

[10] Sweta, V. R., Abhinav, R. P. & Ramesh, A. Role of virtual reality in pain perception of patients following the administration of local anesthesia. *Ann. Maxillofac. Surg.* **9** (1), 110–113 (2019). 31293937 10.4103/ams.ams_263_18 PMC6585215

[11] Steinkraus, K. C. et al. Impact of virtual reality on perioperative pain and anxiety in port implantation under local anaesthesia: A randomized controlled pilot (VIP Trial). *Perioper Med. (Lond)*. **13** (1), 101 (2024). 39390587 10.1186/s13741-024-00454-z PMC11465779

[12] Zengin, S. et al. Effects of music therapy on pain and anxiety in patients undergoing port catheter placement procedure. *Complement. ther. med.* **21** (6), 689–696 (2013). 24280479 10.1016/j.ctim.2013.08.017

[13] Prokopowicz, A., Stanczykiewicz, B. & Uchmanowicz, I. Validation of the numerical anxiety rating scale in postpartum females: A prospective observational study. *Via Med.* **93** (9), 686–694 (2022). 10.5603/GP.a2021.0197 35072220

[14] Jeanne, M., Clément, C., De Jonckheere, J., Logier, R. & Tavernier, B. Variations of the analgesia nociception index during general anaesthesia for laparoscopic abdominal surgery. *J. Clin. Monit. Comput.* **26** (4), 289–294 (2012). 22454275 10.1007/s10877-012-9354-0

[15] Todd, K. H. & Funk, J. P. The minimum clinically important difference in physician-assigned visual analog pain scores. *Acad. Emerg. Med.* **3** (2), 142–146 (1996). 8808375 10.1111/j.1553-2712.1996.tb03402.x

[16] Schaal, N. K. et al. The effects of a music intervention during port catheter placement on anxiety and stress. *Sci. Rep.* **11** (1), 5807 (2021). 33707520 10.1038/s41598-021-85139-z PMC7970967

[17] Machin, D., Campbell, M. J., Tan, S. B. & Tan, S. H. Sample size tables for clinical studies. 61 (Wiley-Blackwell. 2009).

[18] NICE (National Institute for Health and Care Excellence). Guidance on the use of ultrasound locating devices for placing central venous catheters. Technology appraisal guidance. (2002). www.nice.org.uk/guidance/ta49

[19] Morris, R. W. & Whish, D. K. A controlled trial of pain on skin infiltration with local anaesthetics. *Anaesth. Intensive Care*. **12** (2), 113–114 (1984). 6383116 10.1177/0310057X8401200204

[20] McKay, W., Morris, R. & Mushlin, P. Sodium bicarbonate attenuates pain on skin infiltration with lidocaine, with or without epinephrine. *Anesth. Analg*. **66** (6), 572–574 (1987). 3034106

[21] Fleckenstein, F. N. et al. A prospective randomized controlled trial assessing the effect of music on patients’ anxiety in venous catheter placement procedures. *Sci. Rep.* **12** (1), 6922 (2022). 35484279 10.1038/s41598-022-10862-0 PMC9050649

[22] Gold, J. I., SooHoo, M., Laikin, A. M., Lane, A. S. & Klein, M. J. Effect of an immersive virtual reality intervention on pain and anxiety associated with peripheral intravenous catheter placement in the pediatric setting: A randomized clinical trial. *JAMA Netw. Open.* **4** (8), e2122569 (2021). 10.1001/jamanetworkopen.2021.22569 PMC8387848 34432011

[23] Huang, Q., Lin, J., Han, R., Peng, C. & Huang, A. Using virtual reality exposure therapy in pain management: A systematic review and meta-analysis of randomized controlled trials. *Value Health*. **25** (2), 288–301 (2022). 35094802 10.1016/j.jval.2021.04.1285

[24] Menekli, T., Yaprak, B. & Doğan, R. The effect of virtual reality distraction intervention on pain, anxiety, and vital signs of oncology patients undergoing port catheter implantation: A randomized controlled study. *Pain Manag Nurs.* **23** (5), 585–590 (2022). 35367144 10.1016/j.pmn.2022.03.004

[25] Paice, J. A. & Cohen, F. L. Validity of verbally administred numeric rating scale to measure cancer pain intensity. *Cancer Nur s*. **20** (2), 88–93 (1997). 10.1097/00002820-199704000-00002 9145556

[26] Benotsch, E. G., Lutgendorf, S. K., Watson, D., Fick, L. J. & Lang, E. V. Rapid anxiety assessment in medical patients: Evidence for the validity of verbal anxiety ratings. *Ann. Behav. Med.* **22** (3), 199–203 (2000). 11126464 10.1007/BF02895114

[27] Le Guen, M. et al. The analgesia nociception index: a pilot study to evaluation of a new pain parameter during labor. *Int. J. Obstet.* **21** (2), 146–151 (2012). 10.1016/j.ijoa.2012.01.001 22360936

[28] Baroni, D. A., Abreu, L. G., Paiva, S. M. & Costa, L. R. Comparison between analgesia nociception index (ANI) and self-reported measures for diagnosing pain in conscious individuals: a systematic review and meta-analysis. *Sci. Rep.* **12** (1), 2862 (2022). 35190644 10.1038/s41598-022-06993-z PMC8860998

