# Correction: The effects of music and virtual reality on pain and anxiety during central venous port implantation: a randomised clinical trial

**DOI:** 10.1038/s41598-026-53675-1

**Published:** 2026-05-21

**Authors:** Abdelmalek Ghimouz, Sylvain Dureau, Matthieu Carton, Alexandra Gomola, Ziad Fadel, Kim Khanh Thuong, Jane Muret

**Affiliations:** 1 Department of Anaesthesia, Intensive Care, and Pain, Institut Curie, 26, rue d’Ulm, Paris, 75005 France; 2 Departement of Statistics. Institut Curie Paris, 26, rue d’Ulm, Paris, 75005 France; 3 Departement of Anaesthesia, Intensive Care, and Pain. Institut Curie, 35, Rue Dailly, Saint Cloud, 92210 France; 4 Departement of Anaesthesia. Institut Gustave Roussy, 114, Rue Edouard Vaillant, Villejuif, 94800 France

Correction to: *Scientific Reports* 10.1038/s41598-026-42184-w, published online 07 March 2026

The original version of this Article contained errors in the author list. Abdelmalek Ghimouz was incorrectly listed twice and has subsequently been removed. Staff Anesthetist was incorrectly listed as an author with affiliation 5 and has subsequently been removed.

Additionally, Table 5 was incorrectly generated and was split into two parts on page 4 and page 6 in the PDF version of the article. The Table was correct in the HTML version of the Article.

The original Article has been corrected.

